# Seventeen-Year Journey Working With a Master

**DOI:** 10.3389/fimmu.2018.00960

**Published:** 2018-05-02

**Authors:** Jinfang Zhu

**Affiliations:** Molecular and Cellular Immunoregulation Section, Laboratory of Immunology, National Institute of Allergy and Infectious Diseases, National Institutes of Health, Bethesda, MD, United States

**Keywords:** GATA3 transcription factor, T helper cells, innate lymphoid cells, cytokines, T cell differentiation, T cell development

## Abstract

It had been a great honor for me to work with the late Dr. William E. Paul for 17 years in the Laboratory of Immunology (LI) from 1998 until his passing in 2015. He was such a master in the immunology field. Under his outstanding guidance, my research has been focusing on transcriptional regulation of T helper (Th) cell differentiation, especially, on the role of a master transcription factor GATA3 during Th2 cell differentiation. Just as enormous scientific contributions of Dr. Paul (we all call him Bill) to the immunology community are far beyond his serving as the Chief of the LI, GATA3 also plays important roles in different lymphocytes at various developmental stages besides its critical functions in Th2 cells. In this special review dedicated to the memory of Bill, I will summarize the research that I have carried out in Bill’s lab working on GATA3 in the context of related studies by other groups in the field of T cell differentiation and innate lymphoid cell (ILC) development. These include the essential role of GATA3 in regulating Th2/ILC2 differentiation/development and their functions, the critical role of GATA3 during the development of T cells and innate lymphoid cells, and dynamic and quantitative expression of GATA3 in controlling lymphocyte homeostasis and functions.

## Preface

I joined the lab of Dr. William E. Paul (Bill) in 1998 as a postdoctoral fellow soon after I got my Ph.D. degree from the Shanghai Institute of Biochemistry, Chinese Academy of Sciences. Before I arrived in the U.S., Bill and I had already exchanged several emails regarding my potential projects. As a scientist who discovered interleukin (IL)-4, Bill had always been interested in IL-4 signaling and the structure of IL-4 receptor (IL-4R). He initially suggested me to crystallize the intracellular domains of the IL-4Rα chain, but I was more interested in transcriptional regulation of gene expression in lymphocytes, an area no one in Bill’s lab had explored in the past. Bill later asked several other postdocs, who joined his lab after me, to work on IL-4R structure demonstrating his amazing persistence in research and impressive flexibility in mentoring.

Since IL-4 is the critical cytokine for driving type 2 T helper (Th2) cell differentiation (1), my first project started with searching for IL-4-inducible transcription factor(s) during early Th2 cell differentiation using DNA microarray. At the same time, I was working on the cross-regulation of T cell receptor (TCR)- and IL-4-mediated signaling (2) together with Dr. Hua Huang, a senior postdoc in Bill’s lab at that time, who is now a full professor at the National Jewish Health.

My first project ended up with identifying growth factor independent-1 (Gfi-1) as an IL-4-inducible transcription factor, which plays an important role in promoting selective growth of committed Th2 cells (3). Later, Gfi-1 was also reported to suppress Th1, Th17, and Treg cell differentiation and the expression of IL-7 receptor α chain (4–6). The reason why we focused on transcription factors that are induced by IL-4 at *early* stages of Th2 cell differentiation is mainly because, in 1997, Drs. Richard Flavell and Anuradha Ray’s groups had already independently reported that GATA3 is necessary and sufficient for the expression of Th2 cytokines (7, 8).

In our initial report, the effect of Gfi-1 on Th2 cell proliferation was demonstrated by retroviral co-expression of Gfi-1 and GATA3 (3). To further assess whether Gfi-1 indeed plays an important role during Th2 responses under physiological conditions, with the help of Dr. Hua Gu who was a new Principle Investigator in the LI at that time, I started to generate Gfi-1 conditional knockout mice (4). At that time, GATA3 conditional knockout mice were not available either. While I was making Gfi-1 floxed mice, Bill gave me a very important suggestion—why don’t you also prepare GATA3 conditional knockout mice at the same time (9). He said “I believe Gfi-1 is an interesting molecule to further work on, however, GATA3 is probably more important than Gfi-1 for Th2 cells.” Indeed, throughout the 17 years period that I worked with Bill, first on T helper (Th) cell differentiation as a postdoctoral fellow and then on innate lymphoid cell (ILC) development as an independent investigator, I published 15 papers with their titles containing GATA3, but only 5 for Gfi-1. This visionary advice from Bill—always focusing on the most important things—has had a great impact on my research career.

## Introduction

CD4 Th cells orchestrate adaptive immune responses by producing effector cytokines. In order to effectively exert their protective functions during infections, distinct Th subsets are developed to deal with a variety of pathogens (10–12). There are three major Th cell subsets: type 1 T helper (Th1) cells that mainly produce IFN-γ, Th2 cells that produce IL-4, IL-5, and IL-13, and Th17 cells that produce IL-17a and IL-17f (13, 14). Th1 cells are important for immune responses to intracellular bacteria and viruses; Th2 cells are mainly responsible for immunity against helminth infections; whereas Th17 cells are essential for dealing with infections with extracellular bacteria and fungi. Besides their critical roles in mediating protective immunity, Th subsets are also capable of inducing many types of inflammatory responses. While Th2 cells are known to be involved in allergic diseases, Th1 and Th17 cells may cause autoimmunity (12, 15). All the Th effector cells are developed from naïve CD4 T cells when they encounter an antigen/MHCII complex that can be recognized by their antigen-specific TCR. Some naïve CD4 T cells may differentiate into regulatory T cells (Tregs) and they are regarded as peripheral induced Tregs (pTregs); together with thymic-derived Tregs, pTregs regulate the magnitude and duration of a particular immune response in addition to their essential role in maintaining immune tolerance (16–20).

In recent years, a group of non-B non-T lymphocyte-like cells that are capable of producing Th effector cytokines have drawn much attention in the field. These cells are now designated as innate lymphoid cells (ILCs) (21–24). Just like Th cells, there are three major ILC subsets: group 1 ILCs (ILC1s) that mainly produce IFN-γ, ILC2s that produce IL-5 and IL-13, and ILC3s that mainly produce IL-22. Since ILC subsets can produce cytokines known to be effector cytokines of Th cells, ILC and Th subsets of the same group are involved in related type of immune responses in a collaborative manner (25–30). For example, just as Th2 cells, ILC2s are not only involved in immune responses against helminth infections, but also induce allergic inflammation (29, 31–38). Therefore, similar to Th cells serving as professional cytokine-producing cells, ILCs are considered as the innate counterparts of Th cells.

The differentiation of Th1, Th2, and Th17 cells is mainly controlled by cytokine environment during their activation, which induces the expression of lineage-defining transcription factors: T-bet for Th1; GATA3 for Th2; and RORγt for Th17 cells (39). These master regulators are not only essential for the differentiation and functions of Th subsets, but also they are utilized by ILC subsets for their development and functions: T-bet for ILC1s; GATA3 for ILC2s; and RORγt for ILC3s. While T-bet and RORγt are selectively expressed by Th1/ILC1 and Th17/ILC3 subsets, respectively, GATA3 is actually expressed by all the Th and ILC subsets although its expression in Th2 cells and ILC2s is the highest. Furthermore, GATA3 is dynamically expressed during T cell and ILC development. In this mini-review, I will discuss multiple important functions of this master transcription factor in a variety of lymphocytes at different developmental stages (Table 1).

**Table 1:** GATA3 expression and functions in distinct lymphocytes and during T cell/innate lymphoid cell (ILC) development/differentiation.

Cell type or process	GATA3 levels	GATA3 functions	Genes regulated by GATA3	Reference
T helper (Th)2	High	Essential for Th2 cell differentiation and Th2 cytokine production; suppressing the expression of Th1-related genes	Inducing *Il4, Il5, Il13, Areg, Ccr8, Il1rl1, Il7r, Cd69*; suppressing *Ifng, Tbx21, Ccl5, Havcr2*	(7–9, 40, 41, 43, 44, 47, 50, 54, 85)
ILC2	Very high	Essential for ILC2 development and type 2 cytokine production	*Il5, Il13, Areg, Ccr8, Il1rl1, Il7r, Il2ra, Il17rb*	(52–57)
Early T cell development	Intermediate to high	Essential for early T cell development	*Tcr, Cd3*???	(59–61)
CD4 T cell development	Intermediate to high	Essential for CD4 but not CD8 T cell development	*Il7r, Cd3, Th-POK*	(47, 59, 62–64)
PLZF^+^ ILC progenitor	High	Essential for the generation of PLZF/PD-1-expressing ILC progenitors	*Il7r, Id2*???	(54, 65)
LTi progenitor	Low to intermediate	Required for LTi homeostasis and functions but not for development	*Il7r, Lta*???	(54, 65)
Regulatory T cell	Low to high	Defining Treg subsets, modulating Treg functions and stability	*Il7r, Foxp3, Il2ra, Il1rl1, Ccr8*	(47, 66, 68–70)
Natural killer (NK)T cell	Low to high	Defining NKT cell subsets and maintaining homeostasis	*Il7r, Il4*	(74, 75)
CD8 T cell	Low	Homeostasis and memory generation	*Il7r, Myc*	(76)
Th1, Th17, and Tfh cell	Very low to low	Unknown	*Il7r, Ifng, Il4*???	N.A.
ILC3	Intermediate	Essential for the development of NKp46^+^ ILC3s and modulating ILC3 function	Inducing *Il7r, Il22;* suppressing *Rorc*	(58, 65)
ILC1	Intermediate	Homeostasis	*Il7r*???	(26)
NK cell	Low	Maturation	*Ifng*	(72, 73)

*??? refers to likely but not yet confirmed*.

## Critical Role of GATA3 in Th2 Differentiation and Functions

As mentioned earlier, Drs. Flavell and Ray’s groups independently reported that GATA3 is a key transcription factor for inducing Th2 cytokine expression back in 1997 (7, 8). Soon after, Dr. Ken Murphy’s group further showed that enforced expression of retroviral GATA3 induces endogenous GATA3 expression even in cells that were cultured under Th1 polarization conditions (40, 41). However, because GATA3-deficient CD4 T cells were not available at that time, direct evidence to support the essential role of GATA3 during Th2 differentiation particularly *in vivo* was still lacking. Nevertheless, these exciting reports inspired Bill and me to prepare a conditional knockout allele of *Gata3* by the Cre-loxP system (42). By using *Gata3* conditional knockout mice, both Dr. I-Cheng Ho’s group and ours confirmed that GATA3 indeed is the master regulator of Th2 cells (9, 43). In the absence of GATA3, the production of Th2 cytokines is severely impaired, at the same time, IFN-γ production is induced even when the cells are cultured under Th2 conditions (44).

Interleukin-4-mediated STAT6 activation is sufficient to induce GATA3 expression during Th2 cell differentiation (45). Low dose of TCR stimulation can also upregulate GATA3 expression in the absence of IL-4 signaling (46). Indeed, Th2 differentiation may occur *in vivo* in an IL-4-STAT6-independent manner (15). On the other hand, although GATA3 can autoregulate its own expression, GATA3 is not required to induce itself in the presence of IL-4 signaling (47). Nevertheless, IL-4-dependent as well as IL-4-independent Th2 cell differentiation depends on GATA3 both *in vitro* and *in vivo* (9).

Genome-wide analyses of GATA3 binding through ChIP-Seq (chromatin immune-precipitation followed by high throughput sequencing) show that GATA3 binds to the Th2 cytokine locus *Il4/Il13* at multiple sites including sites in the *Il4* intron 2, the *Il13* promoter, and the locus control region within the *Rad50* gene (47). GATA3 also binds to the promoter of the *Il5* genes (48, 49). A major mechanism for GATA3 to induce IL-4 expression is through chromatin remodeling at the *Il4/Il13/Rad50* locus. In mature Th2 cells in which GATA3-mediated epigenetic modifications within the Th2 cytokine locus have already occurred, GATA3 is no longer needed for IL-4 production. However, since the activity of the *Il5* and *Il13* promoters always depends on GATA3, GATA3 deletion at any time completely abolishes IL-5 and IL-13 expression (9). Many other Th2-specific genes as well as long intergenic non-coding RNAs are also directly regulated by GATA3 (50). For example, T1/ST2, the IL-33 receptor encoded by the *Il1rl1* gene, is highly expressed in the most mature Th2 cells and GATA3 binds to the *Il1rl1* gene (47, 50).

## Critical Role of GATA3 in ILC2 Development and Function

When I started my own research group, it had been known that there are a group of non-T non-B innate-like lymphocytes capable of producing type 2 cytokines and that type 2 cytokines produced by CD4 T cells are not essential for host defense (29, 33). Thus, I was very interested in what these cells were and how they developed. We hypothesized that GATA3, the critical factor for type 2 immune responses, may also be functionally important for the generation of type 2 cytokine-producing innate-like lymphocytes. Thus, we started to generate mice with GATA3 deficiency in the hematopoietic system and mice allowing inducible GATA3 deletion.

These innate-like cells are now known as type 2 innate lymphoid cells (ILC2s). Indeed, ILC2s express very high levels of GATA3 and they are highly enriched in the lung, skin, gut, and adipose tissues (21, 31, 35). Strikingly, ILC2s and Th2 cells generated during helminth infection are identical in their transcriptomes (51). Just as its critical function for Th2 cell differentiation, GATA3 is presumably also important for ILC2 development. However, due to its essential role during ILC development in the progenitor stage, which I will discuss later, definitive evidence showing the importance of GATA3 expression for ILC2 development is still lacking. Nevertheless, even in mature ILC2s, deletion of GATA3 results in loss of ILC2 functions (i.e., diminished IL-5 and IL-13 production) and reduced survival of ILC2s (52–57). Genome-wide analysis comparing transcriptomes between wild type ILC2s and GATA3-deficient “ILC2s” indicates that several important genes involved in type 2 immune responses, such as *Il5, Il13, Il1rl1*, and *Ccr8*, etc., are regulated by GATA3 (54). These genes are also regulated by GATA3 in mature Th2 cells, which may explain similar functionalities between ILC2s and Th2 cells.

GATA3 also directly binds to the *Il4/Il13* loci in ILC2s; the pattern of GATA3 binding to the Th2 cytokine locus in ILC2s is very similar to that in Th2 cells (47, 58). It has been reported that GATA3 regulates chromatin remodeling at several Th2-specific gene loci in Th2 cells (47), however, whether GATA3 play a similar role in epigenetic modifications in ILC2s is unknown. GATA3 also regulated the expression of the IL-33 receptor subunit T1/ST2 and IL-25R in ILC2s (47, 54, 58). Therefore, because of the downregulation of IL-33R and IL-25R expression in GATA3-deficient “ILC2s,” these cells fail to respond to either IL-33 or IL-25. GATA3-deficient “ILC2s” also express lower levels of CD25 and IL-7R. Thus, there is a general defect of GATA3-deficient ILC2s in response to multiple cytokines.

## Critical Role of GATA3 in T Cell and ILC Development

Besides its essential function in Th2 cells and ILC2s, GATA3 is also critical for T cell and ILC development at multiple stages (59, 60). GATA3 is important for the generation of T cell progenitors (59, 61). GATA3 is also required for CD4 but not for CD8 T cell development (47, 59, 62–64). Similarly, GATA3 is critical for the development of T helper-like ILCs that express IL-7Rα, but not of NK cells (54). In fact, high levels of GATA3 expression are required for the generation of PLZF/PD-1-expressing non-LTi progenitors but low levels of GATA3 expression are necessary for the function of LTi cells (65). Therefore, helper-like ILCs are considered as the innate counterpart of CD4 Th cells, whereas NK cells resemble innate CD8-like cells, and GATA3 is a master regulator for the development of both innate (ILC) and adaptive (Th) lymphocytes.

## Critical Functions of GATA3 in Tregs

GATA3 is also expressed by Tregs, and under certain circumstances, GATA3 expression may reach high levels, especially when cells receive IL-4 and/or TCR stimulation (66). The expression of some “Th2-related” genes, including *Il1rl1* and *Ccr8*, in Tregs depends on GATA3 (47). GATA3 binds to the *Foxp3* locus at the CNS2 region (67) and such binding may be important for maintaining optimal Foxp3 expression in Tregs (66, 68). Deletion of GATA3 specifically in Treg cells results in uncontrolled systemic Th2 responses in one study (68), however, other studies reported that these GATA3 conditional knockout mice were grossly normal although the GATA3-deficient Tregs showed some abnormal phenotype (66, 69, 70). Interestingly, GATA3 is dynamically expressed by Treg cells (70). Because persistent expression of GATA3 in Tregs at high levels may convert Tregs into Th2 cells (71), dynamic expression of GATA3 may be critical for maintaining Treg phenotype. Together with T-bet, GATA3 also suppresses RORγt expression in Tregs. Therefore, balanced expression of T-bet, GATA3, and RORγt in Foxp3-expressing is critical for Treg-mediated immune regulation (70).

## Important Functions of GATA3 in Other Lymphocytes

GATA3 is expressed by ILC3s at intermediate levels (58). Interestingly, intermediate levels of GATA3 expression are required for regulating the balance between T-bet and RORγt, and thus the development of NKp46^+^ ILC3s (58). GATA3 also regulates IL-22 expression in ILC3s (58). Whether GATA3 regulates the balance between T-bet and RORγt and/or IL-22 production in Th cells requires further investigation. GATA3 is also expression by ILC1s at intermediate levels and GATA3 is required for maintaining ILC1 homeostasis (26, 58). GATA3 is also expressed by NK cells but at low levels. Although GATA3 is not required for the development of conventional NK cells, it affects their maturation and cytokine production (54, 72, 73). GATA3 also affects NKT cell development and functions (74, 75) as well as CD8 T cell homeostasis partly through regulating IL-7Rα expression (76). Furthermore, GATA3 expression is found at low levels in Th1 and Th17 cells; however, its functions in these cells require further investigation.

## Relationship Between GATA3 and Other Important Transcription Factors

During Th2 differentiation, GATA3 can be upregulated by IL-4/STAT6 and/or TCR-mediated signaling (15). However, ILC2 development does not require IL-4/STAT6 signaling. It is possible that Notch signaling plays an important role in GATA3 induction in ILCs. Consistent with this notion, TCF7, a transcription factor induced by Notch signaling, can upregulate GATA3 expression in ILC progenitors (56, 77). What induces/maintains high GATA3 expression in ILC2s is not known.

Although GATA3 plays an essential role in the development and functions of ILC and Th cell subsets, many other transcription factors, including Id2, TCF7, Tox, and Th-POK may form a network with GATA3 in determining cell lineage fates (26, 63, 77, 78). Just as GATA3, Bcl11b is important for the development of T cells and ILC2s (79–84). We have recently reported that GATA3 and Bcl11b form a complex and they co-localized in many enhancer regions within the Th1- and Th2-related genes (85). Interestingly, the GATA3/Bcl11b complex not only suppresses the expression of many Th1-related genes, but it also controls the magnitude of Th2 responses. GATA3 and Bcl11b may have common targets in ILC2s, which requires further investigation.

Several other transcription factors can also interact with GATA3. T-bet interacts with GATA3 and suppresses its function (86, 87). Consequently, T-bet and GATA3 co-bind to many Th1- or Th2-related genes (88–90). T-bet overexpression suppresses GATA3 expression at the transcription level (87). Endogenous expression of T-bet may also inhibit a GATA3-mediated “default” Th2 program during Th1 cell differentiation (90). Interestingly, T-bet is detected in GATA3-expressing cells during helminth infection to limit Th2 responses (91). On the other hand, GATA3 may silence the *Tbx21* gene during Th2 cell differentiation (47). GATA3 may also inhibit Th1 differentiation by suppressing the expression of STAT4 expression as well as Runx3-mediated induction of IFN-γ expression (44, 92), and GATA3 can bind to Runx3 at the protein level.

## Conclusion and Future Directions

Bill was the master of the Laboratory of Immunology at the NIAID, NIH. I had learned tremendously from him through weekly one-on-one meetings throughout the 17-year period working with him. In the earlier era, Bill had also trained many world renowned immunologists, including Drs. Charles Janeway, Mark Davis, Laurie Glimcher, and Ronald Schwartz. Not only Bill had trained many incredible scientists in his lab, but also he had a great impact on our immunology community at the NIH and around the world. Thus, Bill is a true master of immunology. Without him, the NIH immunology interest group has been suffering from a “knockout” phenotype in the past 2 years. We sincerely hope that a master(s) with his/her knowledge and ability equivalent to Bill’s will inspire our community again in the near future.

The master regulator for Th2 cells is GATA3. Just like Bill who contributed to the immunology field in many aspects, GATA3 also plays an essential role during early T cell development, CD4 T cell development as well as ILC development. In mature lymphocytes, ILC2s followed by Th2 cells express the highest levels of GATA3, which is consistent with its critical function in maintaining the functionalities of these type 2 lymphocytes. In other lymphocytes, including Tregs, NKT cells, CD8 T cells, ILC1s, ILC3s, NK cells, and possibly Th1 and Th17 cells, GATA3 may also regulate their homeostasis and functions (Table 1). GATA3-mediated IL-7Rα induction may be a common mechanism through which GATA3 regulates lymphocyte homeostasis; however, this may not fully explain the multifunctions of GATA3 during T cell and ILC development (58, 76).

Because GATA3 is expressed by all T cell and ILC subsets, and its expression varies from cell type to cell type and from stage-to-stage, the functions of GATA3 in different lymphocytes at various developmental and activation stages may be controlled by its expression levels and its interacting partners. Quantitative expression of GATA3 may result in a qualitative effect. To study GATA3 dose effect, a model with a titratable GATA3 expression may be needed to separate the differential roles of GATA3 expressed at high or low levels during the development of T cells and ILCs. Distinct complexes containing GATA3 in different cell types may offer cell-type-specific gene regulation. Thus, identifying GATA3-interacting proteins in different lymphocytes will help us understand the mechanisms of GATA3-mediated gene regulation, which will guide us to obtain deeper insights into the biology of the immune responses in allergic, infectious, autoimmune, and other inflammatory diseases.

We have recently generated a new GATA3 reporter mouse strain through the CRISPR/Cas9 technology by inserting a ZsGreen-T2A cassette into the *Gata3* conditional allele flanked by two LoxP sites. This novel reporter works beautifully: variable GATA3 expression ranging for several logs in GFP intensity is observed in distinct lymphocytes at different developmental stages. We are using this mouse strain in combination with different Cre transgenic lines to study the function of this master regulator in a variety of lymphocytes particularly *in vivo*. We will be happy to share this valuable mouse strain with other labs that are interested in using it, even before its publication, as Bill had taught us the right way to promote science. Although Bill is no longer with us, and I cannot discuss our new exciting results with him anymore, my fascination in studying “master regulators” inspired by Bill will continue endlessly and I believe that is what Bill had hoped for the new generation(s) of immunologists.

## Author Contributions

The author confirms being the sole contributor of this work and approved it for publication.

## Conflict of Interest Statement

The author declares that the research was conducted in the absence of any commercial or financial relationships that could be construed as a potential conflict of interest.
